# Cross-cultural adaptation and validation of the European Portuguese version of the heartland forgiveness scale

**DOI:** 10.1186/s12955-020-01531-9

**Published:** 2020-08-26

**Authors:** Fabio Ikedo, Luisa Castro, Sofia Fraguas, Francisca Rego, Rui Nunes

**Affiliations:** 1 Faculty of Medicine, University of Porto, Alameda Prof. Hernâni Monteiro, 4200-319 Porto, Portugal; 2 Institute for Systems and Computer Engineering, Technology and Science, INESCTEC, Porto, Portugal; 3 Center for Health Technology and Services Research – CINTESIS, University of Porto, Porto, Portugal; 4 University of Algarve, Faro, Portugal

**Keywords:** Forgiveness, Others, Self, Situation, Questionnaire, Psychometrics

## Abstract

**Background:**

Forgiveness is linked with well-being, and social and health research has focused on the role and aspects of forgiveness that has been recently suggested as a phenomenon of public health importance. The Heartland Forgiveness Scale (HFS) was developed gathering three subscales to assess the forgiveness of others, forgiveness of self, and forgiveness of situation. The present study aimed to adapt the HFS into European Portuguese, and investigate its reliability and validity.

**Methods:**

Translation and cross-cultural adaptation were conducted using a multistep forward-back translation process. Internal consistency was assessed by Cronbach’s alpha. Confirmatory factor analysis was conducted to verify that the factor structure is the same as in the original HFS. The short version of the Ruminative Response Scale (RRS) and the Satisfaction with Life Scale (SWLS) were used to examine convergent validity.

**Results:**

A sample of 222 university students, selected through convenience sampling, was used to access the validity of the European Portuguese version of the HFS (EPHFS). Cronbach’s alpha for the European Portuguese HFS subscales were 0.777, 0.814 and 0.816 for Self, Others and Situation, respectively, indicating acceptable reliability. The 3-factor model of the original HFS was replicated in confirmatory factor analysis. As expected by evidence in the literature, positive and statistically significant correlations were found between SWLS and HFS and subscales. RRS showed negative and statistically significant correlations with HFS and subscales.

**Conclusions:**

The European Portuguese version of the HFS presented acceptable internal consistency, construct validity and confirmed the three-factor structure of the original HFS.

## Introduction

Forgiveness has gained an increasing focus in social and health research. Recently, Dr. Tyler J. VanderWeele, professor at Harvard, wrote an editorial called “Is Forgiveness a Public Health Issue?” [1], stating that “because being wronged is common, and the effects of forgiveness on health are substantial, forgiveness should perhaps be viewed as a phenomenon that is not only of moral, theological, and relational significance but of public health importance as well.”

Among different researchers’ definitions, forgiveness can be defined as the framing of a perceived transgression such that the responses to the transgression (and its sequelae) and the transgressor are transformed from negative to positive or neutral. The object of forgiveness (source of a transgression) may be oneself, another person, or a situation viewed as beyond anyone’s control [2].

Forgiving has the potential to reduce stress, breaking cycles of negative affect and rumination [3] . Forgiveness has been positively associated to global mental health [4], linked with longevity and improved physical health [5]. While the ability to forgive others and the self may be beneficial to health, unforgiveness from others is harmful [6]. The tendency to forgive refers to the forgiveness at the level of a global disposition, across relationships and situations [7] . Forgiveness of situations is a component of dispositional forgiveness, being related to, but distinct from, forgiveness of others and self [2]. Forgiveness of situations and self may be important factors for the connection between psychological well-being and forgiveness [2].

The Heartland Forgiveness Scale (HFS) was developed to assess the forgiveness of others, forgiveness of self, and forgiveness of situation using three subscales with six items each [2], on a seven-point Likert scale. Scores range from 18 to 126, with higher scores relating to higher levels of forgiveness. Thompson, et al. (2005) reported that forgiveness was positively correlated to positive affect, cognitive flexibility, and distraction, and negatively correlated to hostility, rumination, and vengeance. Forgiveness predicted four components of psychological well-being: anxiety, depression, anger, and satisfaction with life [2].

The translation and adaptation of HFS has been performed to different languages, including Japanese [8], Taiwanese [9], Turkish [10], and Malay [11] languages. So far, there has been no validated European Portuguese version of the HFS (EPHFS). The aim of the present study was to translate, culturally adapt and validate the HFS for the European Portuguese population.

## Methods

### Cross-cultural adaptation

For the creation of the EPHFS, a methodological study of cultural adaptation and validation was performed. After authorisation from the author of the original scale, the HFS was translated according to a standard criteria for instrument cross-cultural adaptation, following the steps proposed by Beaton et al.: translation, synthesis of translations, back translation, expert committee review and test of the pre-final version [12].

1. Translation: two translations were performed independently from English (source language) to European Portuguese (T1 and T2), by an informed (aware of the concepts being examined in the questionnaire) and an uninformed translator, both bilingual and Portuguese native speakers. Both were professional translators; one being a biologist, and the second one with bachelor in languages and a master degree in translation.
2. Synthesis of the translations: the two translators and one recording observer sat down to synthesize the results of the translations T1 and T2, producing one consensual translation T-12.
3. Back translation: the two back translations (BT1 and BT2) were created, based on the T-12 version, by two professional bilingual (English and Portuguese) translators blinded for the original version, with English as their mother tongue, with master degrees in translation and multilingual information management. They worked independently on their translation process.
4. Expert committee review: translations were reviewed and consolidated (T1, T2, T12, BT1, BT2), together with written reports, reaching consensus on discrepancies, and producing a prefinal version for field testing.
5. Test of the prefinal version: prefinal version was tested in a small group (32 persons, selected by convenience) for comprehensiveness and to check the interpretation and cultural relevance of the translation.
6. Submission of documentation to the developer of the original questionnaire for appraisal of the adaptation process: submission of reports and forms to the developer of the original questionnaire to verify that the recommended stages were followed, and a reasonable translation was achieved.

### Instruments

In order to confirm that the Portuguese HFS represents the related construct, related measures to forgiveness were additionally taken into consideration and applied to the same participants: the Ruminative Response Scale (RRS) and the Satisfaction with Life Scale (SWLS). A short version of the RRS was formed by Treynor, Gonzalez, and Nolen-Hoeksema [13] to remove the items that overlap with items on measures of depressive symptomatology. It consists of 10 items from an original list of 22, using a 4-point Likert-type response scale ranging from 1, almost never, to 4, almost always. The total score can be between 10 and 40, with higher scores corresponding to higher levels of ruminative responses styles. The 10 items are grouped in two subscales: Reflection and Brooding [12]. The original short version of RRS was translated into Portuguese by Dinis A et al. [14]. In their study, the principal component analysis revealed the need to remove item 5 (“I write what I’m thinking about and then I analyze what I wrote.”), for presenting a very low (0.186) communality value [14]. With 9 items, the Portuguese version of RRS showed a good level of internal consistency for each subscale, an adequate temporal stability and good convergent and discriminant validities [14]. In the current study, the Portuguese version of the RRS measure with 9 items was used and the Cronbach’s alpha obtained was 0.665.

The SWLS was developed by Diener et al. [15] to measure the sense of satisfaction with life as a whole. The SWLS is a 5-item self-report unidimensional measure with good psychometric characteristics [16]. Each item can range from 1 (strongly disagree) to 7 (strongly agree) on a 7-point-Likert-type response scale. The total score can be between 5 (low satisfaction) and 35 (high satisfaction). The Portuguese version of the scale was performed by Neto [17]. In the current study, the Cronbach’s alpha obtained for this Portuguese version of the SWLS measure was 0.867.

### Participants

Regarding sample size requirements for factor analysis, rules-of-thumb vary from four to 10 subjects per variable, including a minimum of 100 subjects in order to ensure stability of the variance-covariance matrix [18]. Moreover, other authors recommend a range of 200–300 as appropriate for factor analysis [19, 20]. In line with these recommendations, our sample consisted of 222 students enrolled in two different departments of University of Porto, and selected by convenience sampling, after authorization from the departments. All students inquired agreed to participate. Data was collected by self-administration using paper and pen/pencil in auditorium-style classrooms. The following characteristics were collected from participants: age, gender, degree and year of degree frequency. Confidentiality of data and anonymity were guaranteed to the participants.

### Statistical analysis

To validate the HFS in the European Portuguese population, reliability, construct validity and convergent validity were assessed via confirmatory factor analysis and correlation analysis.

Factor analysis should be applied to determine whether the items form only one factor or dimension (overall scale) or more than one. In cases where there is a clear hypothesis regarding the factor structure, as is the case of HFS for which the factor structure has been already determined [2] and confirmed for other language translations [21], confirmatory factor analysis (CFA) should be used [22].

Specifically, CFA was employed for testing of the hypothesis that the general construct of forgiveness, as measured by the EPHFS, is composed of three separate (although correlated) factors of self, others and situations [2]. In this study, CFA was applied using maximum likelihood and covariance matrices to test the three-factor model of the EPHFS. To account for the multiple aspects of the structural model fit, results should be evaluated using multiple indices [23, 24]. One of the most used absolute index has been the ratio *χ*^2^/*df*, which is said to be less sensitive to sample size than the respective *p*-value. However, there is little statistical or logical rational for it and thus it is not recommended for model fit assessment [24]. Instead, the *χ*^2^ value and the associated *p*-value, should be reported, since model rejection might not be attributed to the large sample size [24]. Regarding relative indices, a value for the Tucker–Lewis index, TLI, and for the comparative fit index, CFI, above 0.90 or 0.95, are generally indicative of acceptable or good model fit, respectively [24–26]. The most popular indices of population discrepancy are the root mean square error of approximation, RMSEA, and the standardized root mean square residual, SRMR. Regarding reference values for these population discrepancy indices, RMSEA should be below 0.08 for a reasonable fit or below 0.05 for a close fit [27], and the SRMR should be below 0.08 to be indicative of good fit [25]. Following the recommendation for using multiple indices, the fit of the models attained from CFA was evaluated using the set of fit indices here described. Finally, and in order to compare non-nested models, the Akaike information criterion (AIC) and the Schwarz’s Bayesian information criterion (BIC) were chosen, where smaller AIC and BIC values indicate a better model.

Internal consistency is one of the simpler reliability metrics and is commonly assessed by the Cronbach’s alpha coefficient, which formula corresponds to the mean of all the possible split-half reliability coefficients of a scale. The most consensual definition of Cronbach’s alpha is the interrelatedness among the items, and which should refer to unidimensional (sub)scales, that is, items measuring the same construct [28]. In this study, a Cronbach’s alpha coefficient between 0.7 and 0.9 was considered to be acceptable [29, 30]. The homogeneity of items can be verified by the analysis of item-total and inter-item correlations, for the items constituting each subdomain or dimension of the scale. The usual rule of thumb is that an item should correlate between 0.3 and 0.7 with the total score of the factor (excluding that item), using Pearson’s coefficient [29]. Additionally, average inter-item correlations for items in the same factor should correlate moderately, between 0.15 and 0.5, to ensure they measure the same construct but not so close as to be almost repetitive [31].

If more than 15% of respondents achieve the lowest or highest possible score, then floor or ceiling effects are present, respectively. The existence of floor or ceiling effects indicates limited content validity [22], hence floor and ceiling effects for each dimension of HFS scale and missing values were also reported for our participants.

For assessing the convergent validity of the new instrument, we examined to which extent the HFS measure of forgiveness is correlated with the RRS and the SWLS (as suggested by HFS authors in [2]). Specifically, the hypotheses to be tested are if SWLS correlates positively with HFS [32] and if RRS correlates negatively with HFS [2]. Some studies also showed a positive correlation between forgiveness and satisfaction with life [33, 34], and a negative association between rumination and forgiveness [35–37]. The normality of the total scores was verified based on skewness and kurtosis values, whose values between − 2 and 2 are assumed as indicative of normal distribution [38]. Following this method, measures from the 3 instruments were assumed as normally distributed, and hence Pearson correlation was used.

Additionally, linear multiple regression analysis was pursued for the construction of a model predicting life satisfaction (as measured with SWLS), having as predictors the factors constituting the Heartland Forgiveness Scale, as suggested for an additional validation of the HFS [2].

IBM SPSS Statistics 24 was used for the statistical analysis and CFA was performed using AMOS 24 [39]. A significance level of 0.05 was pre-defined.

## Results

### Cross-cultural adaptation

The equivalence between the source and EPHFS included semantic, idiomatic, experiential and conceptual aspects. The discrepancies were solved based on consensus of the majority of experts. It was necessary to adapt some of the words related to the 7-point Likert-type response scale to the Portuguese context, such as “Almost Always False of Me: Não Reajo Assim Quase Nunca”.

During stage 4, expert committee review, all 18 items of the T-12 Portuguese version were approved, without modifications. The test of the prefinal version (stage 5) evaluated the degree of understanding, and confirmed that further adjustments were not necessary.

### Participant’s characteristics

From the 222 students enrolled in the study, 155 were women. The median age of participants was 21.0 yr (25th–75th interquartile range, 19–22 yr). Seventy-five participants (33.8%) were from the degree of pharmacy: 66 in the 1st year and 9 frequenting the 2nd year. The remaining 147, in the same university, were frequenting the medicine degree: 21 from the 3rd, 124 from the 4th and 2 students from the 5th year. The completion of the 3 questionnaires took between 5 and 15 min.

### Confirmatory factor analysis

CFA was pursued with first and second-order factors models, following validations published reporting other translations and the original HFS paper [2, 21]. The model with only first-order factors resulted in an initial model with correlated factors of self, other and situation, and not adequate fit: *χ*^2^(132) = 325.046, *p* < 0.001; TLI = 0.833; CFI = 0.856; RMSEA = 0.081 with 90% confidence interval (CI) [0.070, 0.093]; SRMR = 0.077.

In order to increase model fit, covariance between measurement errors from some items of the same factor were added to the model, corresponding to the modification indices with higher values. In order to mitigate type I error, only the modifications with an index above 11 [*χ*^2^(1) = 10.86; *p* = 0.001] were performed. The four modification indices introduced were correlations between items (belonging to the same factor) with the same wording, raising the possibility for the exploration of a deeper model, including methods factors relative to the positive and negative wording of the items, as proposed in [2].

Results for the obtained model revealed reasonable model fit indices for the three-factor model of HFS: *χ*^2^(128) = 243.171, *p* < 0.001; TLI = 0.897; CFI = 0.914; RMSEA = 0.064 with 90% CI [0.051, 0.076]; SRMR = 0.0703; AIC = 329.17 and BIC = 475.49. The significant *p*-value associated with the chi-square test for both versions, means that the exact-fit hypothesis is rejected. However, the relative fit statistics are above the recommended thresholds for acceptance, with the exception of TLI which is close to but less than 0.9 and thus we can accept this model as reasonable, but should also explore another model.

Factor loadings were all statistically significant and their standardized estimates ranged between 0.413 and 0.762. Moreover, results for the estimated correlation coefficients of each factor pair indicated positive correlations with each other (HFS Self and HFS Others, *r* = 0.289; HFS Self and HFS Situation, *r* = 0.804; HFS Others and HFS Situation, *r* = 0.389). These correlation coefficients mean that the three scores change in the same direction, supporting the three-factor model of the HFS in terms of construct validity.

**Fig. 1:**
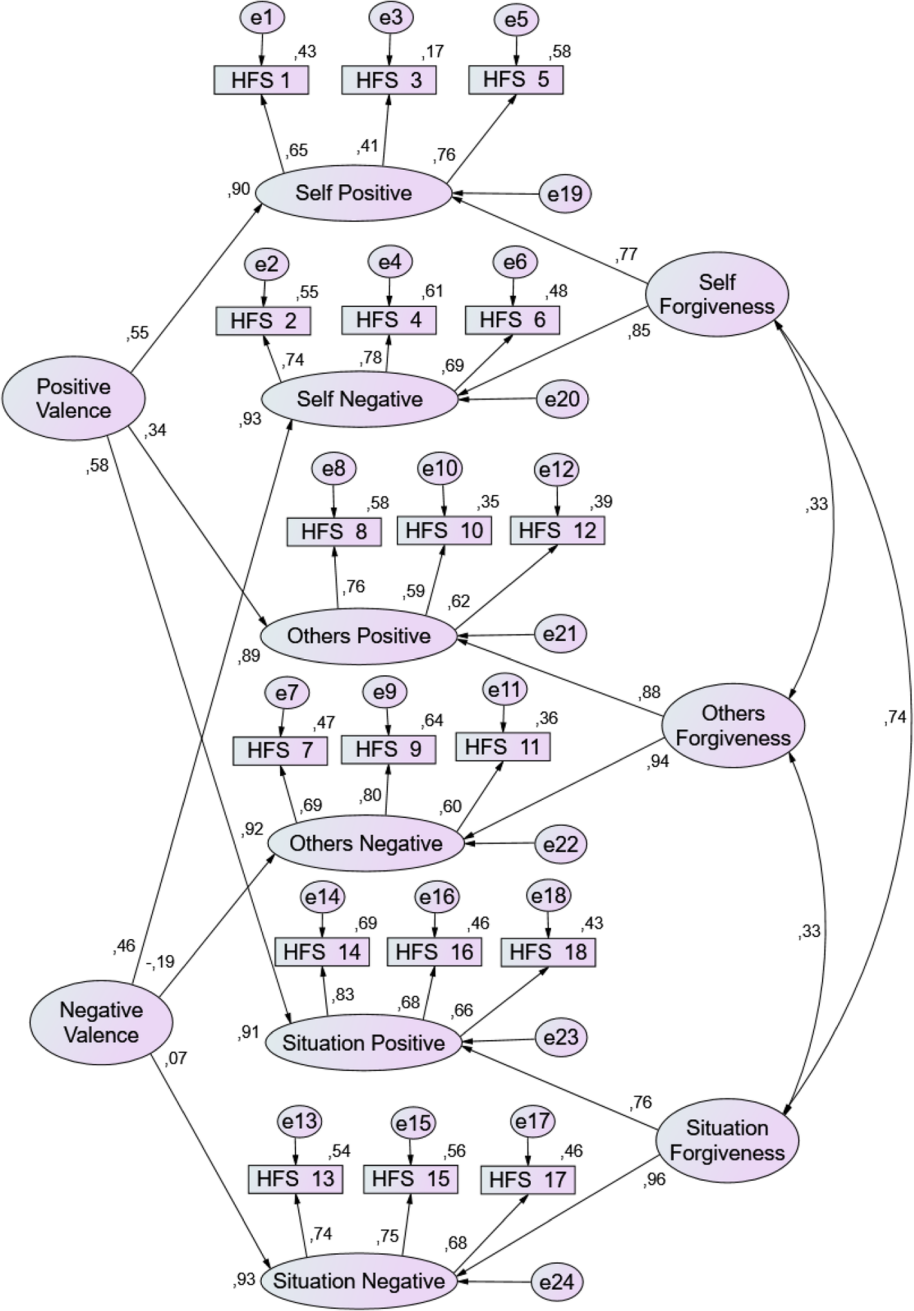
Path diagram for the second order structural factor model of the Heartland Forgiveness Scale (HFS) in students (*N* = 222). Observed HFS items and unobserved factors are indicated in rectangles and larger ovals, respectively. Items error measurements (e1-e18) and residuals for first-order factors (e19-e24) are indicated by small ovals. Values above the items and next to the first-order factors are the model variances, values next to single-headed arrows indicate standardized factor loadings and values next to double-headed arrows represent correlations between factors

The second model for CFA was computed following the suggestion of the original HFS publication [2] of an alternative structure to address the systematic variance due to wording valence while maintaining the distinction between self, others and situation’s forgiveness. The final model structure consists of 6 first-order factors corresponding to negatively and positively worded factors of self, others and situation (see Fig. 1). The second-order layer comprises positive and negative valence factors indicated by the positively and negatively worded first-order factors, respectively. These valence factors, also called methods factors, might account for additional systematic variability in the participants’ responses in an instrument with positive items intermingled with negative ones, as the HFS [40]. Besides these two new factors, the second-order layer comprises also the three forgiveness factors of self, other and situation, each indicated by the corresponding positive and negative valence’s first-order factors. The model was identified following the constraints in the HFS paper [2] except for the imposition of the equality of several factor loadings. Instead, we identified the model by fixing factor loadings to 1, for the first item of each first-order factor (self positive, self negative, other positive, other negative, situation positive and situation negative); fixing the variance of each second-order factor to 1 and setting residual variances to 0.1 for positively and negatively worded first-order factors (except for self positive).

This model resulted in better fit indices, all above the thresholds for adequate model fit: *χ*^2^(125) = 206.84, *p* < 0.001; TLI = 0.925; CFI = 0.939; RMSEA = 0.054 with 90% CI [0.041, 0.067]; SRMR = 0.064; AIC = 298.84 and BIC = 455.37.

For this second model, factor loadings of items were all statistically significant and their standardized estimates ranged between 0.414 and 0.830. Also, results for the estimated correlation coefficients of each factor pair indicated positive correlations with each other but two slightly smaller than in the first model (HFS Self and HFS Others, *r* = 0.326; HFS Self and HFS Situation, *r* = 0.740; HFS Others and HFS Situation, *r* = 0.326).

Besides resulting in better fit indices, the second model should be preferred as it also presents smaller AIC and BIC values than the first model (298.84 and 455.37 vs 329.17 and 475.49, respectively), indicative of a better fit.

### Internal consistency

For EPHFS dimensions of self forgiveness, others forgiveness and situation forgiveness, adequate inter-item correlations were obtained, 0.360, 0.425 and 0.429, respectively, indicating that items must be assessing the same content. Regarding corrected item-total correlations, for each dimension, all items showed adequate item-total correlations (ranging between 0.328 and 0.670), indicating that all items are well correlated with the corresponding factor. Cronbach’s alpha coefficient for EPHFS subscales were 0.777 for HFS Self, 0.814 for HFS Others and 0.816 for HFS Situation, indicating acceptable reliability. Descriptive statistics and internal consistency estimates for the HFS subscales, HFS Total, SWLS and RRS are displayed in Table 1.

**Table 1:** HFS, RRS and SWLS descriptive statistics and Cronbach’s alpha (*N* = 222)

Scale (number of items)	Mean Score	Standard Deviation	Minimum Score	Maximum Score	Cronbach’s Alpha
HFS Self (6)	28.37	5.93	11	41	0.777
HFS Other (6)	28.13	6.18	8	41	0.814
HFS Situation (6)	27.72	6.27	9	42	0.816
HFS Total (18)	84.21	14.14	42	122	0.862
SWLS (5)	25.69	5.91	8	35	0.867
RRS (9)	26.47	4.28	12	36	0.665

Floor and ceiling effects were 0% for the three dimensions with the exception of HFS-Situation which had a 0.9% of participants reaching the higher possible value of the subscale. Together with presenting no missing data, these results provide evidence of the content validity of our EPHFS.

### Convergent validity

Statistically significant and positive correlations were found between the SWLS and the European Portuguese Heartland Forgiveness Scale and subscales, with the HFS Situation subscale achieving the highest correlation, and the HFS Others achieving the lowest (see Table 2). As expected, the RRS showed statistically significant and negative correlations with HFS and subscales, ranging from − 0.361 for HFS Total to − 0.213 for Forgiveness of Others.

**Table 2:** Pearson correlations observed between Heartland Forgiveness Scale (HFS) total score and subscales, Rumination Response Scale (RRS) and Satisfaction with Life Scale (SWLS), in 222 participants

Scale	HFS Self	HFS Others	HFS Situation	HFS Total	SWLS	RRS
HFS Self	1	0.257*	0.600*	0.797*	0.338*	−0.310*
HFS Other	–	1	0.310*	0.682*	0.252*	−0.213*
HFS Situation	–	–	1	0.830*	0.511*	−0.313*
HFS Total	–	–	–	1	0.478*	−0.361*
SWLS	–	–	–	–	1	−0.343*
RRS	–	–	–	–	–	1

*p ≤ 0.001

Another validation was performed regarding the hypothesis raised in the original scale, that dispositional forgiveness (assessed by HFS Situation) would be a significant predictor of measures of physiological wellbeing, even after adjusting for forgiveness of self and others in the regression model [2]. In the present study, SWLS was predicted through a linear multiple regression model, with HFS Self, HFS Others and HFS Situation as predictors. The analyses provided evidence that variable HFS Situation was a significant predictor (β = 0.457; *p* < 0.001) of satisfaction with life, even accounting for the contributions of forgiveness of self and others (β = 0.038; *p* = 0.6 and β = 0.101; *p* = 0.101, respectively), confirming the hypothesis raised.

In agreement with Macaskill [41] we also found a weak positive correlation between age and forgiveness of situations (*r* = 0.159; *p* = 0.018) but not for self or others forgiveness.

## Discussion

We reported the cross-cultural adaptation and validation of the EPHFS. The proposed version was developed according to a standard guideline for instrument cross-cultural adaptation [12]. The Portuguese version of the HFS was easy to understand and deemed to be equivalent to the original version. The consolidated version produced at step 4 (expert committee review) would be the pre-final version but the step 5 (test of the pre-final version) revealed no need of further adjustments, and the pre-final was considered the final version, retaining its equivalence, without redundancies and ambiguities.

Results of this study provide evidence that the EPHFS is a valid and reliable instrument for evaluating forgiveness in the Portuguese population, as defined in the three factor structure proposed by the original HFS version [2]. The EPHFS here described, not only satisfies the expected criteria on internal consistency, convergent validity, with values comparable to those of the original English version, but also confirm its’ three-factorial structure. Reporting zero missing in the participant’s responses to items, this 18-item self-administered questionnaire is easy to administer requiring less than 15 min for completion.

To our knowledge only three published studies have assessed the factor structure of the HFS, based on different target populations [2, 21, 42]. Our student sample results point in the direction of a three-factor model, corroborating the factor structure proposed in the original HFS version, based in a sample of university students [2]. In both models, the 6 first-order factors correspond to negatively and positively worded factors of self, others and situation, while the 5 second-order factors are the main factors of self, others and situation plus the two valence, negative and positive, factors. In our model, self and situation factors were the most correlated (*r* = 0.74) while self and others were the least (*r* = 0.33), supporting also the findings in the original HFS [2]. Also, the loadings for the main factors of self, other and situation were larger than the loadings for the wording valence factors, resembling once again the findings described in Thompson (2005) that the wording valence factors are secondary with respect to the main factors of self, others and situation [2].

In contrast, the Turkish study, also based on a students’ sample, described a first-order model [21] and the Indian employees’ study proposed a different second-order model where the 3 factors of self, others and situation are first-ordered and the second-order is the overall forgiveness [42].

Regarding the convergent validity of HFS, correlations among the EPHFS, the RRS and SWLS, revealed negative correlation between HFS and RRS (high score on the HFS more likely to have lower RRS scores), and positive correlation between HFS and SWLS (high HFS score more likely to get higher scores on the SWLS), both highly significant. Hence, forgiveness was associated with lower rumination and greater life satisfaction, in line with the hypothesis raised from theoretical studies and other translations validation [2, 21].

Besides providing a translated and cross-cultural validated EPHFS, our study contributes to the characterization of the HFS factor structure and also for the measurement of its reliability and validity among Portuguese faculty students. However, some limitations should be acknowledged. A convenience sample of healthcare students from a public Portuguese university was used, thus the sample may not be representative of the entire population of university students in Portugal.

Another limitation refers to the size of the sample, as large samples are recommended for reducing measurement errors and generalizable results to the real population structure [43]. Nevertheless, the number of subjects per item is within recommended numbers, with more than 10 subjects per variable. Future work should entangle larger and more diverse groups of individuals, collecting more demographic characteristics. Another limitation relates to the fact that in our sample, Cronbach’s alpha for the RRS was slightly below the 0.7 cutoff.

The consistency of the EPHFS across time, or test-retest reliability, was not explored in this study, presenting a final limitation. This psychometric property is particularly interesting in clinical practice, for the follow-up of patients, and hence future work should address the temporal consistency of this instrument.

## Conclusion

The EPHFS presented acceptable internal consistency, confirmatory factor analysis of the three-factor structure, and construct validity. Hence, this version can be used in clinical practice and research. The availability of this EPHFS version will enable its use in the Portuguese population promoting forgiveness studies. By the other hand, its equivalence with the original HFS and other language translations may, in the long run, potentiate multi-cultural studies with the gathering of data obtained from equivalent measures of the Heartland Forgiveness Scale. The results here described concerning a new language translation and cultural validation add on previous HFS’s translations and students adaptations pursued, further suggesting that the conceptual structure of HFS is stable in many different countries [42].

## Supplementary information

**Additional file 1. **HFS European Portuguese.

## Data Availability

The datasets used and/or analysed during the current study are available from the corresponding author on reasonable request.

## Abbreviations

AIC: Akaike information criterion
BIC: Schwarz’s Bayesian information criterion
CFA: Confirmatory factor analysis
CFI: Comparative fit index
CI: Confidence interval
EPHFS: European Portuguese version of the HFS
HFS: Heartland Forgiveness Scale
RMSEA: Root mean square error of approximation
RRS: Ruminative Response Scale
SRMR: Standardized root mean square residual
SWLS: Satisfaction with Life Scale
TLI: Tucker–Lewis index
