# Supplementary material for: Cross-cultural adaptation and validation of the European Portuguese version of the heartland forgiveness scale
Source: Health Qual Life Outcomes. 2020 Aug 26;18:289. doi: 10.1186/s12955-020-01531-9 (PMC7449032; doi:10.1186/s12955-020-01531-9)
Supplement: Supplementary file 1 — Additional file 1. HFS European Portuguese. [file 12955_2020_1531_MOESM1_ESM.pdf]

HFS

Instruções:

Ao longo da nossa vida podem ocorrer situações negativas em resultado das nossas ações, ações de outros ou circunstâncias fora do nosso controlo. Durante algum tempo após estes eventos, é possível que surjam sentimentos ou pensamentos negativos sobre nós próprios, sobre os outros ou sobre a situação. Pense em como reage habitualmente a estes eventos negativos. Junto a cada um dos itens seguintes, indique (usando a escala numérica de 1 a 7 abaixo) o número que melhor descreve a sua resposta habitual ao tipo de situações negativas descritas. Não há respostas certas ou erradas. Procure ser o mais sincero possível nas suas respostas.

1	2	3	4	5	6	7
Não Reajo Assim Quase Nunca		Não Reajo Assim Muitas Vezes		Reajo Assim Frequentemente		Reajo Assim Quase Sempre

_____ 1. Embora me sinta mal quando faço uma asneira, com o tempo sou capaz de dar um desconto a mim mesmo (a).

_____ 2. Guardo rancor de mim mesmo (a) por coisas negativas que fiz.

_____ 3. Aprender com as coisas más que fiz ajuda-me a ultrapassá-las.

_____ 4. É muito difícil aceitar-me depois de ter feito asneira.

_____ 5. Com o tempo, torno-me compreensivo (a) para comigo mesmo (a) em relação aos erros que cometi.

_____ 6. Não paro de me criticar por coisas negativas que senti, pensei, disse ou fiz.

_____ 7. Continuo a castigar uma pessoa que fez algo que considero errado.

_____ 8. Com o tempo, torno-me compreensivo (a) para com os outros em relação aos erros que cometeram.

_____ 9. Continuo a ser duro (a) com outros que me magoaram.

_____ 10. Embora outros me tenham magoado no passado, acabei por conseguir vê-los como boas pessoas.

_____ 11. Se outros me maltratam, continuo a pensar mal deles.

_____ 12. Quando alguém me desilude, acabo por conseguir ultrapassar a situação.

_____ 13. Quando as coisas correm mal por razões que não podem ser controladas, fico preso (a) em pensamentos negativos sobre elas.

_____ 14. Com o tempo, consigo ser compreensivo (a) para com as circunstâncias negativas da minha vida.

_____ 15. Se circunstâncias incontroláveis na minha vida me deixam desapontado (a), continuo a pensar negativamente sobre elas.

_____ 16. Acabo por aceitar as situações negativas da minha vida.

_____ 17. Tenho muita dificuldade em aceitar situações negativas que não são culpa de ninguém.

_____ 18. Acabo por me libertar dos pensamentos negativos sobre circunstâncias negativas que estão fora do controlo de todos.
